# Towards a framework for analyzing determinants of performance of community health workers in malaria prevention and control: a systematic review

**DOI:** 10.1186/s12960-018-0284-x

**Published:** 2018-05-08

**Authors:** Helen Mwiinga Chipukuma, Joseph Mumba Zulu, Choolwe Jacobs, Gershom Chongwe, Mumbi Chola, Hikabasa Halwiindi, Jessy Zgambo, Charles Michelo

**Affiliations:** 1 Department of Health Policy Unit, School of Public Health, University of Zambia, P.O. Box 50110, Lusaka, Zambia; 2 Department of Health Promotion Unit, School of Public Health, University of Zambia, P.O. Box 50110, Lusaka, Zambia; 3 Department of Epidemiology and Biostatistics Unit, School of Public Health, University of Zambia, P.O. Box 50110, Lusaka, Zambia; 4 Department of Environmental Health Unit, School of Public Health, University of Zambia, P.O. Box 50110, Lusaka, Zambia

**Keywords:** Community health worker, Performance, Evaluation, Malaria, Assessment, Utilization, Implementation, Policy

## Abstract

**Background:**

Community health workers (CHWs) are an important human resource in improving coverage of and success to interventions aimed at reducing malaria incidence. Evidence suggests that the performance of CHWs in malaria programs varies in different contexts. However, comprehensive frameworks, based on systematic reviews, to guide the analysis of determinants of performance of CHWs in malaria prevention and control programs are lacking.

**Methods:**

We systematically searched Google Scholar, Science Direct, and PubMed including reference lists that had English language publications. We included 16 full text articles that evaluated CHW performance in malaria control. Search terms were used and studies that had performance as an outcome of interest attributed to community-based interventions done by CHWs were included.

**Results:**

Sixteen studies were included in the final review and were mostly on malaria Rapid Diagnosis and Treatment, as well as adherence to referral guidelines. Factors determining performance and effective implementation of CHW malaria programs included health system factors such as nature of training of CHWs; type of supervision including feedback process; availability of stocks, supplies, and job aids; nature of work environment and reporting systems; availability of financial resources and transport systems; types of remuneration; health staff confidence in CHWs; and workload. In addition, community dynamics such as nature of community connectedness and support from the community and utilization of services by the community also influenced performance. Furthermore, community health worker characteristics such marital status, sex, and CHW confidence levels also shaped CHW performance.

**Conclusions:**

Effectively analyzing and promoting the performance of CHWs in malaria prevention and control programs may require adopting a framework that considers health systems and community factors as well as community health worker characteristics.

## Background

Globally, malaria has declined in incidence by 37% and mortality rate by 60% between 2000 and 2015 [1]. Community interventions through Community Health Workers (CHWs), who are a link between the community and the health facility, have contributed to this reduction [2–4]. The World Health Organization (WHO), in attempting to move toward elimination of malaria, has come up with a strategy that has three main building blocks which are to ensure universal access to malaria prevention, diagnosis, and treatment, to accelerate efforts toward elimination of malaria and attainment of malaria-free status, and to transform malaria surveillance into a core intervention [2]. These building blocks can be best achieved through Primary Health Care (PHC) using the CHW as key actors in the strategy, which most countries adopted after the 1978 Declaration of Alma-Ata [3, 4].

Community health workers are men and women with basic literacy and numeracy levels, chosen by the community and trained to deal with individual and community health problems while working in close relationship with the formal health care system [5–7]. This review focused on trained CHWs who work as community malaria agents (CMAs) carrying out malaria prevention and control interventions in the community.

Studies have shown that CHW performance can help reduce morbidity and mortality in resource constrained settings [3, 7, 8]. The CHW performance is context-specific [7], and there is little evidence on what specific factors have contributed to effective implementation of the CHW strategy in malaria interventions. Community health workers provide cost-effective and sustainable ways of delivering malaria control interventions in the community. These interventions include conducting rapid diagnostic tests, malaria treatment, community sensitization for IRS, and distribution of insecticide treated nets (ITNs) [9]. Despite great efforts to combat it, the threat of resurgent malaria is present across different settings. Resurgence has in part been attributed to non-cooperation of communities in control initiatives [10]. Awareness of this threat and the development of systems to minimize resurgence are key to further progress in malaria control [10]. This systematic review was done to gather evidence of CHW performance evaluations in malaria and how they were measured, highlighting determinants of their performance in malaria prevention and control programs.

## Methods

### Search strategy

Google Scholar, Science Direct, and PubMed were searched from September to October 2017. We also searched for and retrieved articles from reference lists using different terms for community health workers. Search terms included “Community Health Workers” OR “Community Malaria Agents” OR “Community Based Volunteers” OR “Village Health Workers” OR “Community Health Aides” OR “Community Health Agents” “Health Extension Workers” OR “Health Surveillance Assistants” OR “Community Medicine Distributors” AND malaria AND “evaluation” OR “assessment” OR “performance” AND “Sub-Sahara Africa”. Alternative terms for performance were evaluation and assessment.

### Inclusion criteria

The search was limited to English peer-reviewed publications of observational and interventional studies with quantitative and mixed methods analysis. The review included publications addressing evaluation or performance assessment outcomes of CHWs working in malaria preventive and curative programs in the sub-Sahara Africa. Only publications from 2000 to 2016 were included, as this marked the period in which there was a distinct drop in malaria cases and deaths attributed to CHW efforts. Studies that evaluated CHW programs which covered effectiveness of CHWs or responsiveness of individual CHWs, community and program implementers to the malaria CHW strategy with regards to sensitization, surveillance, diagnosis, treatment, follow-up of malaria cases, environmental management, and described a factor promoting or affecting malaria CHW program outcomes were included.

### Study selection and quality assessment

Four reviewers from a team of eight independently assessed titles and abstracts. The other four reviewers read the full texts of identified peer-reviewed articles to evaluate potential eligibility. Another reviewer’s opinion was sought in case of persisting disagreements until consensus was reached. The study selection was guided by the PRISMA guidelines, and quality of these studies was assessed using the critical appraisal skills program (CASP 2015) to ensure methodically proven reliable evidence-based studies in the review. The quality criteria we used were as follows:

- Whether the research questions or objectives were clearly stated?
- Whether the approach was appropriate for the research question?
- Whether the study context was clearly described?
- Whether the role of the researcher was clearly described?
- Whether the sampling method was clearly described?
- Whether the sampling strategy was appropriate for the research question?
- Whether the method of data collection was clearly described?
- Whether the data collection method was appropriate to the research question?
- Whether the method of analysis was clearly described?
- Whether the analysis was appropriate for the research question?
- Whether the claims made are supported by sufficient evidence?

All studies included in the review focused on malaria CHW strategy with clearly stated objectives addressing the question under review and highlighting factors that affected performance positively or negatively. The sampling methods were well explained, and the data collection methods were described clearly with appropriate analyses whose claims were supported by evidence in all the articles.

Data was extracted onto a data extraction form created in Microsoft Excel to assess information on key study aspects such as the objectives, designs, sample, performance measurement tool, and results. The data extraction form also contained a description of the intervention of study and the outcome measures (Table 1).

**Table 1:** Summary of included studies

S/N	Author	Title	Aim	Evaluation tool	Study design	Key results-PICO
1	Kelly et al. (2001) Kenya	Community health worker performance in the management of multiple childhood illnesses: Siaya District, Kenya, 1997–2001	To characterize community health worker (CHW) performance using an algorithm for managing common childhood illnesses	An algorithm for managing common childhood illnesses	Cross-sectional studie followed up in 1998, 1999, and 2001	Participants- 100, 108, and 114 CHWs Intervention- Community case management Comparison- Baseline and end line data Outcome- Performance deficiencies were found in the management of sick children by CHWs, although care was not consistently poor
2	Chanda et al. (2011) Zambia	Community case management of malaria with RDT	To evaluate the effectiveness of using CHWs as delivery points for ACT and RDTs in the home management of malaria in two districts in Zambia	Direct observation-Practical	A mixed method prospective study	Participants- CHW and facility staff Intervention- CCM with RDT and ACT Comparison- None Outcome- Community case management of malaria by CHWs using RDTs and ACT is feasible, acceptable by the communities, and efficient including referral of cases for further management at the health facility
3	Kawakatsu et al. (2015) Kenya	Individual and contextual factors associated with CHW performance in Nyanza province, Kenya- A multilevel analysis	To assess the CHWs’ performance in Western Kenya and describe determinants of that performance	Generated by three indicators: reporting rate, health knowledge, and household coverage	Cross-sectional survey	Participants- CHW Intervention- CHW strategy Comparison- None Outcome- Performance varied according to indicators. The significant factors associated with the CHWs’ performance were some demographic factors, supervisions received and health knowledge
4	MOH-Rwanda (2009) Rwanda	Community case management-Evaluation report of CHW performance—Kigali Rwanda	To analyze CHWs performance in order to use early lessons learned to inform the program expansion	Observation of the CHW demonstrating key competencies	Cross-sectional study with retrospective review of the records	Participant- 35 CHWs Intervention- CCM Comparison- Baseline and end line data Outcome- CHWs performance are strongly linked to the level of simplicity of the management tools, the quality of the training they received—which should be a competency-based training and the quality of the mentoring they received on site after the training
5	Bagonza et al. (2014) Uganda	Performance of CHW in ICCM- Uganda	To assess factors influencing performance of CHWs managing malaria, pneumonia, and diarrhea under the Integrated Community Case Management (ICCM) program in Wakiso District, central Uganda	Composite scores based on the core activities of CHWs under the ICCM program	A cross-sectional study for quantitative methods	Participants- 336 CHW Intervention- ICCM Comparison- None Outcome- Only one in every five CHWs performed optimally under the ICCM program
6	Kalyango et al. (2012) Uganda	Performance of community health workers under integrated community case management of childhood illnesses in eastern Uganda	Compared performance of CHWs managing malaria and pneumonia with performance of CHWs managing malaria alone in and the factors influencing performance	Knowledge tests, case scenarios of sick children, review of CHWs’ registers, and observation of CHWs	Cross-sectional with mixed methods study (June–July 2011)	Participants- CHW-125 CHW Intervention- ICCM by CHWs Comparison- CHWs managing malaria alone and malaria and pneumonia Outcome- The factors perceived to influence CHWs’ performance were community support and confidence, continued training, availability of drugs and other necessary supplies, and cooperation from formal health workers CHWs providing dual-illness management handled malaria cases as well as CHWs providing single-illness management, and also performed reasonably well in the management of pneumonia
7	Chinbuah et al. (2013) Ghana	Assessment of the adherence of community health workers to dosing and referral guidelines for the management of fever in children under 5 years: a study in Dangme West District, Ghana	Assessed CHWs’ adherence to dosing and referral guidelines	IMCI guidelines, data collection forms, and analysis of records	A cluster-randomized, stepped-wedge, controlled, open trial	Participants- 660 CHWs, 100 children (12–59 months)/14 clusters Intervention- Antimalarial versus an antimalarial plus an antibiotic for the treatment of fever among children aged 2–59 months Comparison- Antimalarial only vs. antimalarial with antibiotic Outcome- Adherence to dosing guidelines was high. Adherence to referral guidelines was inadequate
8	Druetz et al. (2015) Burkina Faso	Utilization of CHW for malaria treatment: Results from a three-year panel study in the districts of Kaya and Zorgho, Burkina Faso	To assess effectiveness or feasibility/acceptability of ICCM under real-world conditions of implementation at national scale	Questionnaires	Cross-sectional household panel study from 2011 to 2013	Participants- Children under 60 months of age were enrolled in the panel (*N* = 2237) Intervention- CHW strategy for malaria treatment Comparison- Urban and rural Outcome- In urban areas less than 1% of sick children consulted a CHW while 1–9% in rural areas. CHW rarely used
9	Perez et al. (2009) Mali	The role of community health workers in improving child health programs in Mali	To assess the performance of CHWs in the promotion of basic child health services in rural Mali	Questionnaires	Community-based cross-sectional survey	Participants- CHW (72) and caregivers households) Intervention- CHW intervention Comparison- Households with and without CHW visits Outcome- A positive influence of CHWs on family health practices
10	Mubi et al. (2011) Tanzania	Malaria Rapid Testing by community health workers is effective and safe for targeting malaria treatment: Randomized cross-over trial in Tanzania	Assessing the impact of rapid malaria diagnostic tests (RDTs) by community health workers (CHWs) on provision of artemisinin-based combination therapy (ACT) and health outcome in fever patients	Direct observation-Practical	Randomized cross-over trial	Participants- Twenty-two CHWs and 2930 fever patients Intervention- RDTs by CHWs Comparison- None Outcome- CHWs adhered to the RDT results in 1411 of 1457 patients
11	Yeboah-Antwi et al. (2010) Zambia	Community case management of fever due to malaria and pneumonia in children under five in Zambia: A cluster randomized controlled trial	To assess the effectiveness and feasibility of using CHWs to manage non-severe pneumonia and uncomplicated malaria with the aid of rapid diagnostic tests (RDTs)	Checking of the registers and records, direct observation to interpret the results of RDTs	Cluster randomized controlled trial that compared cross-sectional household surveys	Participants- CHW and children—3125 with 18 CHWs and 2084 with 19 CHWs in control children Intervention- CHWs performed RDTs, treated test-positive children with AL Control CHWs did not perform RDTs, treated all febrile children with AL Comparison- Intervention and control arm—two models for community-based management of malaria in children Baseline and post-study Outcome-primary outcomes were the use of AL in children with fever and early Secondary outcome was the proportion ofchildren who experienced treatment failure
12	Searle et al. (2016) Zambia	Evaluation of the operational challenges in implementing reactive screen-and-treat and implications for malaria elimination in a region of low transmission in southern Zambia	To evaluate operational challenges in implementing reactive screen-and-treat	Records, ground truth evaluation of community health worker performance	Serial cross-sectional surveys	Participants- CHW Intervention- Test and treat Comparison- None Outcome- Poor coverage**—**with limited resources, coverage and diagnostic tools, reactive screen-and-treat will likely not be sufficient to achieve malaria elimination in this setting
13	Kisia et al. (2012) Kenya	Factors associated with utilization of community health workers in improving access to malaria treatment among children in Kenya	Examines factors associated with utilization of CHWs in improving access to malaria treatment among children under five years of age by women caregivers in two malaria endemic districts in Kenya	Conducted using a standardized malaria indicator questionnaire	A cross-sectional household survey	P-Households- Baseline (*n* = 1187) and one year later at end line assessment (*n* = 1374) I- CHW under CCM C- Before intervention and after intervention O- Increase in utilization of CHWs as source of advice/treatment for child fevers from 2% at baseline to 35% at end line, accompanied by a decline in care-seeking from government facilities and other sources including shops
14	Wanduru et al. (2016) Uganda	The performance of community health workers in the management of multiple childhood infectious diseases in Lira, northern Uganda	Assess the performance of CHWs in managing malaria, pneumonia, and diarrhea in the rural district of Lira, in northern Uganda	Combining scores from knowledge assessment and case management	Mixed methods cross-sectional study	P- 428 CHWs, 7 key informants I- CHW management of multiple illnesses C- None O- Low performance in malaria management-education level, duration of training, number of households allocated to each CHW, and supervision frequency were significant
15	Nsona et al. (2012) Malawi	Scaling up integrated community case management of childhood illness: update from Malawi	To provide an overview of the implementation of CCM in Malawi	Program records and Health Management Information System (HMIS) reports from the Integrated Management of Childhood Illness (IMCI) unit in the Ministry of Health (MOH)	Cross-sectional study	P- Program managers and health surveillance Assistants (HSA) I- ICCM by HSA C- Baseline implementation data and 3-year post implementation data O- ICCM strategy has the potential to achieve the government’s goal of universal coverage of key child health interventions because of strong MOH support for both policy and practice
16	Banek et al. (2015) Uganda	Community case management of malaria: exploring support, capacity, and motivation of community medicine distributors in Uganda	To understand the level of support available and the capacity and motivation of community health workers to deliver these expanded services	Questionnaires to gather information about the CMDs’ work experience and to assess knowledge of fever case management, and in-depth interviews	Mixed methods cross-sectional design	P- 100 CMDs interviews and 35 for full transcription and analysis. I- Home-based management of fever (HBMF) program, (ICCM) by CMDs C- None O- CMDs demotivated and faced multiple challenges including high patient load, limited knowledge and supervision, lack of compensation, limited drugs and supplies, and unrealistic expectations of community members

### Data analysis and synthesis

Data from the selected articles was analyzed using NVivo version 10 software. The analysis involved identification, coding, and exploration of relationships of themes within data. A code list was developed which comprised of broad themes collectively agreed upon by the research team members after preliminary reading of abstracts. The code list was later modified to accommodate emergent themes and imported into NVivo. Data from the included articles was coded in the respective nodes by two separate researchers including the principal investigator to allow for inter-coder reliability tests. Where there were discrepancies, the researchers discussed until consensus was reached on how information could be coded. Code reports were for identification of specific factors affecting performance of CHW in malaria programs. Descriptive analysis of the contents of all papers reviewed was conducted per category (thematic coding) and new (sub) categories deriving from the literature were added to the framework (Table 2).

**Table 2:** Determinants of performance in malaria programs

S/N	Factor	Description of determinant	Studies
**Community health worker (CHW) characteristics**			
1	Demographic factors	Female CHWs performed better than their male counterparts, married CHWs gave a higher performance than others, having fewer household duties encourages CHWs to work more actively and reduces the dropout rate, longer work experience, good educational status, availability of supporters for household chores, and appropriate sanitation practices	Kawakatsu et al. 2015; Bagonza et al. 2014; Wanduru et al. 2016
2	CHW confidence and competence	CHWs may have lacked confidence in the guidelines, particularly, the ability of CHWs to obtain an accurate history of convulsions. CHW able to do RDT confidently and effectively on the other hand	Kelly et al. 2001; Mubi et al. 2011
**Community factors**			
1	Community factors	Mobilization of communities by the local leaders and confidence of the community in medicines used, lack of community appreciation for age restrictions of children treated, poor community participation, poor cooperation from caregivers, social prestige, community support in terms of feedback and rewards, training institute, poor performance of basic household health practices	Kalyango et al. 2012; Druetz et al. 2015; Chinbuah et al. 2013; Kawakatsu et al. 2015; Perez et al. 2009; Banek et al. 2015; Nsona et al. 2012
2	CHW service utilization	CHWs rarely use for malaria interventions and only poor household using them in mostly in rural	Druetz et al. 2015; Kisia et al. 2012; Kelly et al. 2001; Yeboah-Antwi et al. 2010
**Health system factors**			
1	Feedback	Some CHWs did not receive timely feedback from their supervisors. Community support in the form of feedback and rewards	Bagonza et al. 2014; Kalyango et al. 2012
2	Training	Adequately continuously trained and appropriately resourced CHWs are really motivated to perform their tasks like interpreting RDTs, and give treatment for Malaria. clearly defined roles for CHAs and supervisors	Kalyango et al. 2012; Chanda et al. 2011; MOH-Rwanda 2009; Yeboah-Antwi et al. 2010; Druetz et al. 2015; Perez et al. 2009; Nsona et al. 2012; Wanduru et al. 2016
3	Stocks and supplies	The need for continuous supplies of drugs and stocks is cardinal for enhancing success of CHWs for malaria	Kelly et al. 2001; Chanda et al. 2011; Kalyango et al. 2012; Searle et al. 2016; Druetz et al. 2015; Perez et al. 2009; Chinbuah et al. 2013; Banek et al. 2015; Nsona et al. 2012
4	Job aids	Complexity of guidelines was an important reason for deficiencies managing sick children. These should be in local language possibly	Chinbuah et al. 2013; Kelly et al. 2001; Nsona et al. 2012
5	Supervision	High quality support supervision from supervisors from formal health system who should have adequate health knowledge to conduct routine supervisions to sustain a high performance is necessary to improve the performance of CHWs in malaria interventions	Bagonza et al. 2014; Kawakatsu et al. 2015; Perez et al. 2009; Kelly et al. 2001; Druetz et al. 2015; Chinbuah et al. 2013; Nsona et al. 2012; Wanduru et al. 2016
6	Funding	CHW performance is hard to achieve and to maintain without sufficient consideration for funding	Druetz et al. 2015; Banek et al. 2015; Nsona et al. 2012
7	Transport	Distance and lack of transport refund affects their performance	Kalyango et al. 2012; Perez et al. 2009
8	Remuneration/motivation	Lack of incentives demotivates them and CHWs asking for consideration	Druetz et al. 2015; Perez et al. 2009; Kalyango et al. 2012; Chinbuah et al. 2013; Banek et al. 2015; Nsona et al. 2012; Wanduru et al. 2016
9	Health professional support	CHWs work not trusted by the health professional staff	Kalyango et al. 2012
10	Workload	CHWs performed poorly due to large population coverage and multiple tasks	Yeboah-Antwi et al. 2010; Kalyango et al. 2012; Searle et al. 2016; Perez et al. 2009; Kelly et al. 2001; Bagonza et al. 2014; Wanduru et al. 2016
11	Evaluation environment	Evaluated in a different setting other than area of usual practice	Kelly et al. 2001
12	Reporting	CHW performance may have been underestimated because a failure to document was interpreted as an error—missing data	Kelly et al. 2001; Nsona et al. 2012
13	Program coordination	Poor CHW program coordination at all levels affects performance negatively	Nsona et al. 2012

## Results

**Fig. 1:**
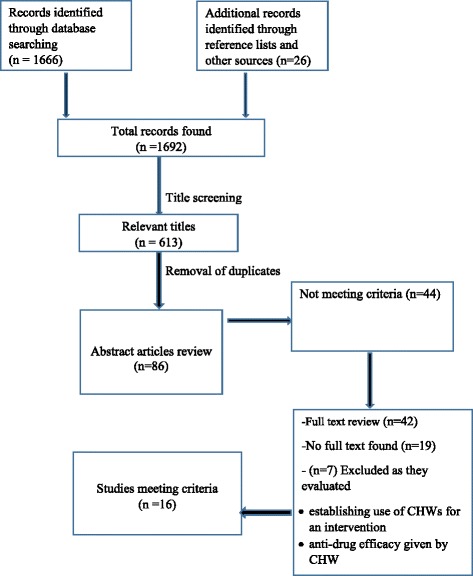
Flow chart: search results

The search resulted in a total of *N = 1692* results appearing on the databases of which *n = 251* were from Google Scholar, *n = 796* from Science Direct, *n = 619* from PubMed, and *n = 26* from reference lists of some articles. After title screening, *n = 613* articles remained and *n = 527* duplicates were removed leaving *n = 86* articles for full abstract assessment. Abstracts not reporting the outcome of interest were *n = 44*, and *n = 42* were considered for full text review. No full texts were found for *n = 19* articles, and *n = 7* were excluded as they were establishing use of CHW for an intervention while others were establishing efficacy of antimalarial drug given by CHWs. Some papers were excluded because they were not from sub-Saharan Africa and were published outside the review period. All the search results were managed in Endnote referencing software (Thomson Reuters, Philadelphia, USA) and the final 16 articles have been reported in this systematic review (Fig. 1).

### Study characteristics

A total of 16 studies are included in this review, 5 of which were from East Africa, 4 from Central Africa, 3 from West Africa, and 4 from Southern Africa. Countries represented were Uganda with four studies [11–14], Kenya with three [15–17], one from Rwanda [18], one from Tanzania [19], one from Burkina Faso [20], one from Ghana [21], one from Mali [22], one from Malawi [23], and three from Zambia [24–27]. Twelve of the studies had a cross-sectional design [11–18, 20, 22, 23, 25], one was a mixed methods prospective study [24], one was a cluster randomized control trial [26], one was a randomized crossover trial [22], and one randomized control stepped wedge trial [21] (Table 1).

### Outcome of malaria evaluations

Most studies included in this review were evaluating diagnosis and treatment in community case management representing 88% of the studies that evaluated performance [11–14, 16, 18, 21–24, 26, 28]. Performance outcomes in malaria varied in different contexts, though generally poor. For instance, some deficiencies were found in management of sick children in Integrated Community Case Management (ICCM) [11, 16] despite reports that CHWs were able to use rapid diagnostic test (RDT) and artemisinin-based combination therapy (ACT) [19, 24, 26] and that the strategy has potential for universal coverage [23]. Different factors perceived to influence performance were grouped into individual CHW characteristics, health system factors, and community factors (Table 2). Only two studies evaluated adherence which was generally high while referral practices were poor [21, 24]. Two of the included studies assessed general performance in relation to the outcome indicators based on general scheduled activities [15, 18]. Table 1 gives a summary of included studies indicating the study designs, method of evaluation, sample characteristics, intervention, comparison, and the outcome of the study (PICO).

### Factors shaping the performance of community health workers in malaria prevention interventions

This section outlines the factors that shape the performance of CHWs in programs aimed at managing malaria. The factors have been grouped into three major headings: CHW characteristics, health systems factors, and community factors. Table 2 gives details of different factors influencing performance of CHW in malaria interventions.

### Community health worker characteristics

#### Demographic factors

Being a female CHW was associated with performance as some female community members who were found home during CHW visits felt freer to be served by a fellow female CHW [11]. Being married also showed to have an influence on performance due to availability of supporters within the household of the CHWs to enable him or her perform her malaria-related duties [11, 15]. Attainment of secondary-level education positively influenced performance as CHWs would easily read and write monthly reports [14]. Longer work experience also positively influenced performance as it provided an opportunity to receive effective training, supervision, and incentives and to build a confidential relationship with community members [15].

#### Competence and confidence levels

How well the CHWs were conversant with the guidelines for Integrated Management of Childhood Illnesses (IMCI) guidelines was vital in promoting work performance. CHWs who lacked knowledge in the guidelines could not perform certain tasks such as obtaining an accurate history of convulsions in successive evaluations due to different terms used that may have meant shivering or startles hence recording poor performance [16]. Competence and confidence in undertaking tasks was considered in four studies investigating CHW ability to use RDT for malaria diagnosis and treatment in the community [19, 24, 26, 28]. Results showed that CHWs were able to manage malaria fevers correctly and that RDTs in the hands of CHWs may safely improve early and well-targeted ACT treatment in malaria patients at community level in Africa. Following guidelines was a key determinant of competence and confidence in one’s responsibilities [16].

### Health system factors

#### Availability of financial resources

Poor performance in Community Case Management (CCM) was partly due to inadequate funding which negatively affected CHW work motivation as CHWs could not meet the set targets [13, 20, 23]. In Burkina Faso, for instance, less than 10% of the overall funding to scale up interventions against malaria was allocated to CCM, despite the ambitious objective to have CHWs manage 80% of all simple malaria cases. This goes to confirm the statement, “there has been no serious attempt to globalize investment in CHWs as a strategy to combat malaria” [20].

#### Motivation

Eight studies emphasized on the importance of motivation for enhancing CHW performance [12, 13, 16, 20–23, 27]. Motivation of CHWs with adequate support from the health system through the introduction of financial incentives and remuneration were among key factors to improve the work of CHWs in rural-urban area communities [13, 20, 22]. Rewards such as t-shirts, blood pressure checks, and transport fares remunerated during review meetings were also found to have greater influence on CHW performance [12, 21]. The CHWs were, in several programs, employed on a voluntary basis or as a regular employee without a fixed monthly salary [22]. Studies have recommended that local health authorities and community representatives clarify how CHWs will be employed. This has a bearing on their motivation to perform tasks, feasibility of sustaining community health activities, as well as affecting efforts of decreasing dropout rates of CHWs to avoid additional costs of replacing them [15, 22]. In Malawi, the stability and community earned respect of the Health Surveillance Assistants (HSAs) has been attributed to their recognition as formal members of the health work force. Provision of adequate housing to HSAs in hard-to-reach areas through village health committees also helped in improving performance, as well as raising the social status of the CHWs and their families [13, 16, 23].

#### Transport systems

Community health workers may live in places too far for community members to consult them [20]. Lack of transport for following up treated children and delivering malaria tasks is another hindrance to work performance [12, 22]. In most cases, CHWs cover vast areas, which complicate their ability to effectively follow up children in rural communities [12, 22].

#### Training

Nine studies indicated the importance of training of CHW prior to implementing malaria interventions [12, 14, 18, 20, 22–24, 26, 27]. These studies show that adequately, continuously trained and appropriately resourced CHWs are motivated to perform their tasks such as interpreting RDTs, and giving treatment for malaria [26]. This is in addition to having clear definition and an understanding of staff responsibilities at the health posts [27]. Evidence shows that CHW trainings must be short as they perform better with only introduction to basic concepts in relation to managing diseases and are to take 2–3 days. Longer training may lead to information overload and thus result in less retention [23]. Performance is strongly linked to the level of simplicity of the management tools including the quality of training received. The training should be a competency-based training focused on exercises, demonstration, role play, video projection, case studies, and practices. Performance is also linked to the quality of mentoring they received on site after training [18]. The CHWs were trained only to prescribe antimalarial drugs to confirmed malaria cases and refer other non-malaria patients to the nearest health facility for further management. They also received training in filling in registers, managing drug supplies, counseling caregivers of children, and adverse reaction monitoring [12, 24]. Some settings also showed that CHWs faced obstacles to performance due to lack of CHW training or regular refresher courses [20, 27].

#### Supervision process

High quality support and supervision by supervisors from the formal health system is necessary to improve CHW performance. The ability of the supervisor to effectively translate knowledge acquired from the supervisory course into proper definition of tasks at the health posts was essential in enhancing performance [11, 15, 16, 20–23, 27].

Insufficient supervision affected the performance of CHWs. For instance, in some supervisory records, fewer than half of CHWs who performed poorly had received one-on-one clinical supervision at a health facility in the past year and a few others had not received any supervision in the previous 6 months [16, 20, 22]. The CHWs who had met with their supervisors in the last 3 months were likely to perform better than those who had not [27], and performance improvement was noticed when trained field supervisors provided additional support and fortnight supervision [21].

Another aspect of supervision is feedback processes. Two studies emphasized the importance of providing feedback in shaping work performance in the ICCM [11, 12, 27]. However, nearly a half of the CHWs did not receive timely feedback from their supervisors [11]. Regular supervision and CHW involvement in meetings were important because it provided opportunities for interactions, clarifications, and receiving feedback, which can act as a social glue for holding staff together [27].

#### CHW program coordination

Strong links between community programs and the formal health system are required to ensure appropriate training and supervision, and adequate remuneration of community health workers but these components still remain weak [23]. There must be clear leadership at central (Ministry of Health), provincial, and district levels and an understanding of stakeholders’ roles and responsibilities under WHO guidelines. These are to monitor and evaluate programs and develop appropriate evaluation tools, reporting tools, and registers. Quality of care assessment of CHWs performance is important as well as district-community-clinic review meetings to strengthen implementation of CHW programs for malaria [23].

#### Reporting System

Only two studies [16, 23] highlighted issues concerning reporting CHW performance. The two studies indicated that performance may have been underestimated because failure to document could have been interpreted as an error-missing data [16]. Performance and efficiency may be improved with innovations such as provision of mobile phones to CHWs. This would facilitate contact and SMS-based reporting and for logistics management information systems to strengthen use and management of medicines and other supplies [16, 23].

#### Availability of stocks and supplies

The need for continuous supply of stock is cardinal for motivation and enhancing success of CHWs [12, 16, 20–25]. Following training, the health facility should provide logistics and supplies for the CHWs’ routine work [24, 25]. Some studies indicated poor performance due to CHW program factors such as irregular supply or lack of materials to enable them to perform their work at night and during rainy weather [12]. Insufficient RDTs reported by over 50% of CHWs sometimes were as a consequence of high number of cases during the peak malaria season [25] and difficulty in anticipating additional quantities of RDTs needed to conduct reactive case detection. This highlights the need for guidance on quantification of medicines to District Medical Officers [23]. Provision of medicines and supplies to CHWs by supervisors during their monthly visits may help to alleviate stock outs [23]. Other hindrances included the lack of aides such as watches and treatment guidelines. The treatment guidelines were reported to be long and that they had ambiguities in the clinical algorithm, and they also showed discrepancies between the drug dosing chart and the algorithm [16].

#### Nature of health professional support

The CHWs expressed concern about criticism from health professional staff for unnecessarily referring a child to a health facility. As a result, when CHWs are in doubt, they may tend to choose a less severe classification or decide not to recommend referral even when they assign a severe classification [16]. Sometimes CHWs would refer a patient to the health center but they would not get the needed attention as the health workers displayed mistrust of the referral instead of attending to the patient [12].

#### Nature of the evaluation environment

The unfamiliar hospital setting also made some CHWs nervous, leading to errors [16]. Although they were instructed to provide treatment as though they were in their home community, some CHWs did not follow the procedures correctly in the hospital environment and could not also document the need for referral [16]. As a result the evaluation which was conducted in the hospital setting showed that some referral data were missing [16].

#### Amount of workload

Seven studies discussed workload issues as affecting work performance [11, 12, 14, 16, 22, 25, 26]. The CHWs performed poorly due to large population coverage and multiple tasks. They tend to get overwhelmed with so many programs when they are to be apportioned only a reasonable amount of work. Workload was mentioned as a key determinant of performance in qualitative analysis [14]. For example, poor basic household health practices put pressure on CHWs for regular follow-up [11, 12, 22]. Thus follow-ups were difficult due to high number of cases during peak malaria season [22, 25]. In contrast, in terms of multitasking, two studies concluded that with appropriate training, adequate supervision, provision of drugs and necessary supplies, community support, and feedback provision from the formal health system, CHWs can provide integrated treatment for malaria and pneumonia, and that additional tasks do not reduce the quality of malaria community case management [11, 12, 16, 26].

### Community factors

#### Support from the community and community connectedness

Eight studies reported community factors and their role in shaping work performance [12, 13, 15, 20–23, 27]. Community ownership through dialog before introduction of the services is vital for successful participation in the malaria programs, for instance, formation of village health committees (VHC) and engagement of community leaders to manage the VHC [23]. Factors affecting performance included mobilization of communities by local leaders and confidence of the community in medicines used by CHWs. Community support in the form of feedback and rewards such as in-kind incentives from community members was found to have greater influence on CHW performance than that from the health system [12, 27]. Some settings have faced some of the most common obstacles to performance, such as caregivers resisting to be being referred [20, 21] and unrealistic expectations of caregivers [13]. Social prestige and community support are the important community level factors associated with CHW performance [15].

#### Utilization of services provided by CHWs

Another factor affecting performance related to how the community is utilizing the services provided by CHWs [16, 17, 20, 26]. Certain skills such as referral by CHWs in some areas could not be meaningfully evaluated because of the small number of clients [16, 20]. People chose a different source of treatment other than CHWs because of various reasons, but distance was statistically significant [20, 26]. Poor and smaller community size of less than 100 households were important characteristics influencing the utilization of CHW services as community case management was offered for free and that CHWs provided prompt treatment at household level [17]. This suggests that issues related to implementation fidelity, acceptability, or feasibility have undermined the effectiveness of CHW programs in countries like Burkina Faso [20].

## Discussion

This systematic review has found that CHW performance was evaluated differently in different malaria endemic settings without a standard evaluation tool. Most evaluations were competency based, focusing mainly on RDT, treatment, and referral services. This is similar to a study by Yasauko (2010) that assessed the quality of service of village malaria workers in Cambodia. In this study, village health workers (VHW) focused on diagnosis and treatment, ignoring other community malaria preventive roles. The study recommended the need to cover other aspects of malaria control in order to further strengthen community-based malaria control [29]. The roles need to include an integrated approach covering surveillance, communication, vector control, and environmental management including evaluating implementation of these instilled CHW skills in terms of quality and a measure of the intended purpose of the intervention to the community as CHW performance cannot only be evaluated through a skill but also by an outcome of the intervention [30].

**Fig. 2:**
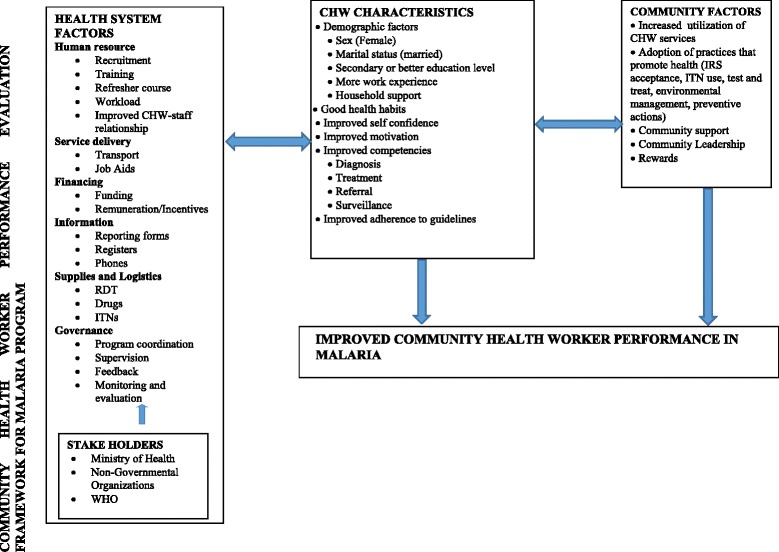
Framework for factors surrounding improved malaria CHW performance-Health system factors, community health worker characteristics and community factors all interlinking

Using evidence of determinants of performance in malaria interventions, we have proposed a performance framework which shows the elements that are vital for determining performance in community malaria programs. The major components are health system factors, CHW characteristics, and community factors (Fig. 2).

Our findings indicate that all elements of the WHO building blocks within the health system interlink in a complex manner and may not flow in a linear manner all the time. These have a significant influence on implementation of CHW programs in malaria interventions and their performance. The individual CHW characteristics are modified by the health system factors to create an enabling environment for individual CHWs, while community factors also play a role in shaping overall performance (Fig. 2). This means that CHWs cannot implement community malaria interventions without support from the formal health system. According to the findings, the malaria CHW strategy needs considerations for CHW characteristics like selecting at least more female CHWs, married CHWs, those with longer experience in community service, secondary level educational status, those with availability of support for household chores, and those with good sanitation practices. These characteristics should be taken into account when developing the selection criteria for CHW community-based malaria programs. The community leadership must be involved and consulted in selecting which CHWs may be engaged as this may assist in obtaining positive response from the community to the malaria interventions [7]. In addition, while WHO recommends that CHWs should receive remuneration, most CHWs are working on voluntary basis and their working terms are not made clear to them except for example the Malawian government that has salaried the health surveillance assistants including providing accommodation, a system that has led to improvement and scaling up of ICCM [23].

Improvement of CHW-professional staff relationship is important for improving CHW confidence, as it provides a level of motivation for a well-coordinated CHW program. Improved cognitive reflections exhibited in CHWs are part of performance measurements worth considering [30]. The CHW competencies in diagnosis, treatment, referral, and surveillance are a measure of individual CHW performance in malaria, but output is also dependent on training received, refresher courses, supervision, logistics, and supplies. Evidence indicates that competence evaluations through observations may have better results if evaluations are done within the CHW work environment [16]. A systematic review by Smith et al. 2014 on the effectiveness of strategies to improve community case management (CCM) of malaria reports that CHWs are able to provide good quality malaria care, including performing procedures such as rapid diagnostic tests with appropriate training, clear guidelines, and regular supportive supervision [31]. Evidence from a systematic review on the impact and implementation of supervision suggests that improving supervision quality has a greater impact than increasing frequency of supervision alone [32].

There is a need to strengthen CHW program coordination among stakeholders such as health ministries and nongovernmental organizations that play a big role in strategy or intervention implementation at district level. These stakeholders have a direct influence on the health system factors related to production of guidelines, registers, checklists, reporting tools, and evaluation tools of which studies revealed not to have had reporting or standard evaluation tools for malaria programs [11, 12, 15]. However, this review found that there are few or no reporting or standard evaluation tools for malaria programs though an innovation to improve the information system through use of phones for reporting has been effected in some countries like Zambia [33].

Motivated CHW may help encourage the community to adopt practices that promote health through witnessing visible changes in the community by CHW efforts as they are a link to the community [7]. Remuneration, availability of supplies, and relevant infrastructure have been found to be important demotivating factors for health workers [34]. To avoid demotivating CHW and health workers alike, sufficient remuneration; supplies of RDT, drugs, and ITNs; and job aids need to be consistent, including relevant infrastructure [23, 27, 34]. One study indicates that there is no serious attempt to globalize investment in CHWs as a strategy to combat malaria [35] hence funding allocation and remuneration for CHW programs should be increased if malaria elimination is to be achieved [20, 23, 35].

In this review, CHWs performed poorly due to increased workload as they have a large population coverage and perform multiple tasks. There is need for scaling up of these malaria CHW interventions and promoting continued use of CHWs in national programs as an important human resource that contributes to long-term impact of interventions [4, 35]. Integrating malaria control activities for CHWs as a holistic package is critical in the fight to eradicate malaria [27]. This entails continued recruitment and training to help reduce the workload and increase coverage. A systematic review assessing the most effective approach to delivering malaria treatment in developing countries recommended that CHWs roles should be recognized, integrated, and expanded as they were effective despite challenges met [35]. Adequate training and refresher courses are vital as knowledge on malaria epidemiology and vector ecology is essential in promoting integration and expansion of CHW practice [36]. This integrated approach may help foster the new WHO pillar strategy that has three main building blocks which are to ensure universal access to malaria prevention, diagnosis, and treatment, to accelerate efforts toward elimination of malaria and attainment of malaria-free status, and to transform malaria surveillance into a core intervention [2]. However, integrating and multitasking with other health programs needs total health system support as priority for CHWs to perform as an important cadre in delivery of primary health care services to the community [4].

The CHWs serve the community, and community feedback is therefore vital as this influences performance. Studies included in the review indicated poor utilization of CHW services [20] and that only the rural poor are utilizing their services [17]. Utilization of CHWs can be improved through engagement of community leadership who may influence the community to support the CHW malaria strategy through rewards [17] and adopting practices that prevent and control malaria. The CHW program is intended not only to improve intervention coverage but also to improve surveillance and reduce congestion in health facilities even in urban areas which are densely populated [3].

### Strengths and limitations

The strength of this review is that it shows actual determinants of CHW performance specific to community malaria interventions in the sub-Saharan Africa region. It adds value to current literature, as it included both qualitative and quantitative studies and was able to explore perceptions. Additionally, this review reflects performance evaluation areas that have been previously poorly explored with respect to CHW interventions in malaria programs. Emphasis has been on improved competencies in diagnosis, treatment, and referral, concentrating on factors within the CHW sphere that enhance performance but are a measure of individual performance in itself. Despite these findings, this systematic review may have been limited by language restriction to English only and also by CHWs having different names in different settings, possibly leading to exclusion of some eligible studies.

## Conclusion

This review has shown that health system factors, community factors, and CHW characteristics were important factors that affect CHW performance with the health system factors being the most important. These CHWs are available and willing to serve the community but a workable environment for them has not been well established in many health systems. Factors affecting performance highlighted in this review should be taken into account by policymakers during designing, implementation, and adjusting of CHW programs with consideration that these factors interlink. Using evidence of performance determinants in malaria programs from various studies, we therefore suggest that a CHW performance framework developed from these studies could guide designing, implementation, and evaluation of community-based malaria prevention programs.

## Abbreviations

ACT: Artemisinin-based combination therapy
CBV: Community-based volunteers
CHA: Community health aides OR Community health agents
CHW: Community health workers
CMA: Community malaria agents
CMD: Community medicine distributor
HEW: Health extension workers
HSA: Health surveillance assistants
ICCM: Integrated community case management
IRS: Indoor residual spraying
ITN: Insecticide treated net
PHC: Primary health care
RDT: Rapid diagnostic test
VHW: Village health workers
